# Clinical and Molecular Differences between 4-Year-Old Monozygous Male Twins Mosaic for Normal, Premutation and Fragile X Full Mutation Alleles

**DOI:** 10.3390/genes10040279

**Published:** 2019-04-05

**Authors:** Alison Pandelache, Emma K Baker, Solange M. Aliaga, Marta Arpone, Robin Forbes, Zornitza Stark, David Francis, David E. Godler

**Affiliations:** 1 Victorian Clinical Genetics Services and Murdoch Children’s Research Institute, Royal Children’s Hospital, Melbourne, VIC 3052, Australia; alison.pandelache@vcgs.org.au (A.P.); robin.forbes@vcgs.org.au (R.F.); zornitza.stark@vcgs.org.au (Z.S.); david.francis@vcgs.org.au (D.F.); 2 Diagnosis and Development, Murdoch Children’s Research Institute, Royal Children’s Hospital, Melbourne, VIC 3052, Australia; emma.baker@mcri.edu.au (E.K.B.); solange.aliagavera@mcri.edu.au (S.M.A.); marta.arpone@mcri.edu.au (M.A.); 3 Faculty of Medicine, Dentistry and Health Sciences, Department of Paediatrics, University of Melbourne, Parkville, VIC 3052, Australia; 4 Brain and Mind, Murdoch Children’s Research Institute, Royal Children’s Hospital, Melbourne, VIC 3052, Australia

**Keywords:** fragile-X syndrome, *FMR1* gene, methylation, mosaicism, expansion, retraction, monozygous twins

## Abstract

This study describes monozygotic (MZ) male twins with fragile X syndrome (FXS), mosaic for normal size (NS: <44 CGGs), premutation (PM: 55–199 CGG) and full mutation (FM alleles ≥ 200) alleles, with autism. At 4 years of age chromosomal microarray confirmed monozygosity with both twins showing an XY sex complement. Normal size (30 CGG), PM (99 CGG) and FM (388–1632 CGGs) alleles were detected in Twin 1 (T1) by standard polymerase chain reaction (PCR) and Southern blot testing, while only PM (99 CGG) and FM (672–1025) alleles were identified in Twin 2 (T2). At ~5 years, T2 had greater intellectual impairments with a full scale IQ (FSIQ) of 55 and verbal IQ (VIQ) of 59, compared to FSIQ of 62 and VIQ of 78 for T1. This was consistent with the quantitative *FMR1* methylation testing, revealing 10% higher methylation at 80% for T2; suggesting that less active unmethylated alleles were present in T2 as compared to T1. AmplideX methylation PCR also identified partial methylation, including an unmethylated NS allele in T2, undetected by standard testing. In conclusion, this report demonstrates significant differences in intellectual functioning between the MZ twins mosaic for NS, PM and FM alleles with partial *FMR1* promoter methylation.

## 1. Introduction

Fragile X syndrome (FXS) is the most common single-gene cause of intellectual disability (ID) and comorbid autism, with an estimated incidence of 1 in 4000 males and 1 in 8000 females [1]. It is complex and heterogeneous in both clinical phenotype and epigenotype, caused by an expansion of a trinucleotide CGG repeat in the *FMR1* gene. The *FMR1* allele sizes are variable and can be classified into distinct repeat sizes that include normal size (NS: <44 CGGs), grey zone (GZ: 45–54 CGGs) and premutation (PM: 55–199 CGGs). Expansions ≥ 200 CGG are known as full mutation (FM) and are invariably FXS affected [2,3]. The FM expansions are usually associated with subsequent DNA methylation of the promoter region, and consequently silencing of the *FMR1* transcription resulting in loss of its protein product (FMRP) [4,5,6,7]. This silencing occurs at 11 weeks of gestation, causing the loss of the protein product FMRP, which is essential for normal neurodevelopment [3]. While the NS, GZ and PM alleles have an unmethylated *FMR1* promoter and express FMRP, the PM alleles have been associated with adult onset disorders including fragile X-associated primary ovarian insufficiency (FXPOI) and the fragile X-associated tremor/ataxia syndrome (FXTAS) [8]. PMs are more common than FM alleles, found 1 in 300 to 1 in 800 males, and 1 in 200 to 1 in 370 females in the general population [9].

Premutation and FM alleles are unstable and the risk of further expansion when maternally inherited. In contrast, it has been proposed that retractions can occur postzygotically [10]. This is used to counsel couples undergoing prenatal diagnosis of the condition. Prenatal testing for FXS typically involves foetal sex determination by karyotype and or Flurorescent In Situ Hybridization (FISH), followed by sizing of the *FMR1* allele either through Southern blot and CGG PCR sizing or through Triplet Repeat Primed PCR and linkage techniques [10]. Presence of mosaicism due to somatic retraction is of particular concern for prenatal testing, where FM alleles are present in a small proportion of cells due to tissue and/or CGG size mosaicism [11,12]. The most extreme example of such retraction has recently been observed in a twin pregnancy, where a couple chose not to terminate, finding a fully retracted seemingly functional normal size allele postnatally in absence of a FM in the male twin [12].

This study describes the first case of 4 years old monozygotic (MZ) twins affected with FXS resulting from an expansion of maternal 109 CGG allele, with discordant phenotypes and CGG size and methylation mosaicism for NS, PM and FM alleles.

## 2. Materials and Methods

### 2.1. Ethics Approval

The clinical and molecular assessment follow-up was conducted as part of the FREE FX study, approved by The Royal Children’s Hospital Research and Ethics Committee (HREC #33066).

### 2.2. Sample Processing

**Figure 1:**
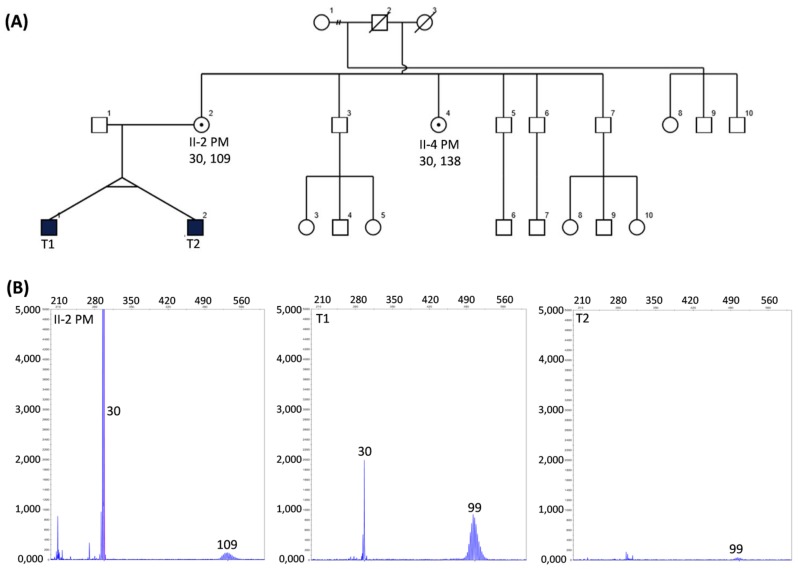
(**A**) Pedigree of the studied family, with squares and circles symbolising males and females, respectively. Black and while shapes indicate affected and nonaffected statuses, respectively. A small black circle in a white shape indicates carrier status. CGG size is indicated by numbers below each participant’s ID; (**B**) Capillary electrophoresis sizing using standard PCR, as previously described [14], with allele sizes indicated by numbers superimposed on the panels.

Saliva samples were collected using the Oragene^®^ DNA Self-Collection Kit (DNA Genotek, Ottawa, Canada) from both twins, their PM mother and maternal aunt (Figure 1). A single saliva swab (DNA Genotek, Global) collected orally per participant, was processed as per manufacturer’s instructions. DNA was extracted using QIAsymphony DSP DNA extraction Kit (Qiagen, Hilden, Germany) at the Victorian Clinical Genetics Services (VCGS), with DNA quality and concentration assessed by NanoDrop 2000 spectrophotometer. Newborn Blood Spots (NBS) from the MZ twins were retrieved from the VCGS NBS repositories as part of the FREE FX study. One 3-mm punch from each NBS disk was placed in a 1.5-mL Eppendorf tube using the Wallac DBS Puncher (Perkin Elmer, Waltham, MA, USA), and incubated in 55 μL in salt lysis buffer (Murdoch Children’s Research Institute, Melbourne, Australia) for 15 min in a heat block for cell lysis, degradation of proteins and release of the DNA, as previously described [13].

### 2.3. CGG Sizing

First-line *FMR1* testing on the saliva DNA at VCGS involved a validated PCR amplification assay with precision of ±2 repeat error measurement and detection limit of 170 CGG repeats in males and 130 CGG repeats in females using Gene Mapper software [14]. Second-line confirmatory testing involved Southern blot analysis, as previously described [15].

### 2.4. FMR1 Methylation Analysis

Extracted DNA samples from NBS lysates were transferred into 96-well plates to be treated with sodium bisulphite. EZ DNA Methylation-Gold™ kit (Zymo research, Irvine, CA, USA) was used to bisulphite convert each sample in two separate reactions, with each conversion analysed in duplicate reactions using the EpiTYPER system and MS-QMA. *FMR1* methylation analysis was performed using Methylation Sensitive-Quantitative Melt Analysis (MS-QMA) and the EpiTYPER system targeting the Fragile X-Related Epigenetic Element 2 (FREE2), as previously described [13]. CGG sizing and mean methylation at two *HpaII* sites, 5′ and 3′ of the CGG expansion, using saliva DNA, was also performed using the AmplideX^®^™ *FMR1* mPCR Kit, as per manufacturer’s instructions (Asuragen, Austin, TX, USA) [16].

### 2.5. Microarray SNP Testing

As part of further investigations, an Illumina CytoSNP300K microarray analysis was performed as per manufacturer’s instructions (Illumina, San Diego, CA, USA) on saliva DNA from the PM mother and both twins, to exclude a second X chromosome and to confirm MZ inheritance. 

### 2.6. Follow-Up Neuropsychological Assessments

The twins were assessed with the Wechsler Preschool and Primary Scale of Intelligence-3rd Edition (WPPSI-III) [17]. This is a standardised test of intelligence that assesses a child’s cognitive development in the domains of verbal IQ, performance IQ, processing speed and full scale IQ. The twins were also assessed using the Autism Diagnostic Observation Schedule-2nd Edition (ADOS-2) [18] for semistructured assessment of autism features. Separate calibrated severity scores (CSS) were derived for the social affect (SA CSS), repetitive and restricted behaviour (RRB CSS) and overall (ADOS CSS) domains of the ADOS-2 [19]. Assessments were undertaken by a member of the research team who had undertaken ADOS-2 for research training and had demonstrated >80% coding reliability across all five modules. 

## 3. Results

### 3.1. Clinical History

A 38-year-old mother with no known family history of FXS or ID gave birth to naturally conceived MZ male twins at 36 weeks of gestation through Caesarean section. T1 required supplementary oxygen at birth, while T2 had micrognathia and a cleft palate (Pierre–Robin sequence, PRS). Single-nucleotide polymorphisms (SNP) microarray testing was subsequently requested for T2 to investigate the cause of PRS. At 40 years of age the mother experienced early menopause. At four years of age, a family paediatrician referred the MZ twins to the genetic clinic at VCGS and fragile X and repeat SNP microarray testing was subsequently requested on saliva samples to investigate a potential genetic cause of ID/ASD in both MZ twins. The same testing was also performed in the mother using a saliva sample. At this time (42 years of age) the mother was suffering from extreme anxiety requiring psychological support and medication. A saliva sample was also collected from the mother’s sister and was tested for fragile X, as she was experiencing symptoms of FXTAS, with a referral to a neurologist recommended.

### 3.2. Initial Diagostic Testing

**Figure 2:**
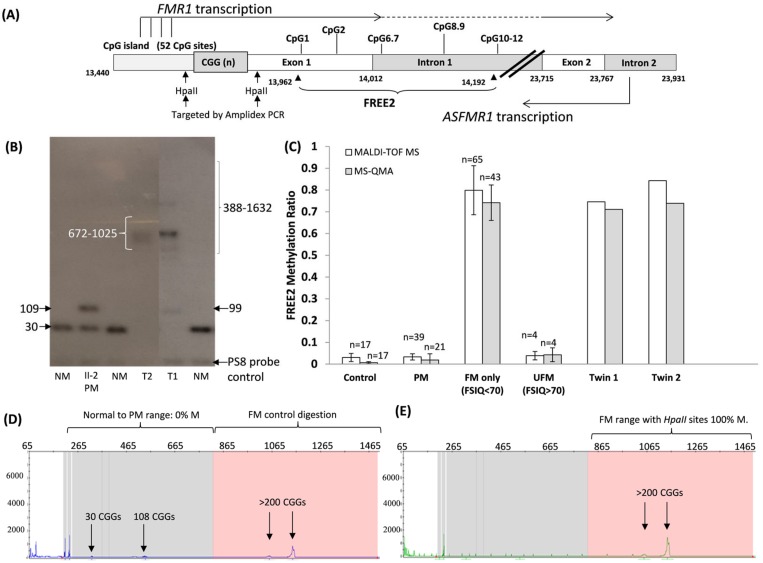
(**A**) Organization of the *FMR1* 5′ region including the CGG expansion (sequence numbering from GenBank L29074 L38501) in relation to *FMR1* and *ASFMR1* transcription start sites (the broken lines indicated spliced out regions), Fragile X-Related Epigenetic Element 2 (FREE2), the *FMR1* CpG island and two methylation sensitive restriction sites *HpaII* sites targeted by AmplideX methylation PCR. The FREE2 region is located downstream of the CGG expansion, at the exon 1/intron 1 boundary and includes 12 CpG sites. (**B**) Southern blot analysis of the DNA sample in question from the two twins (T1 and T2) and their mother (PM II-2) with numbers next to arrows indicating CGG repeat length. Comparator DNA sized using standard CGG PCR [14] from normal allele size males (NM) was included. (**C**) Mean methylation output ration of CpG sites located within the FREE2 region. Assessed using Matrix-Assisted Laser Desorption/Ionisation-Time-Of-Flight Mass Spectrometry (MALDI-TOF MS) and Methylation Sensitive-Quantitative Melt Analysis (MS-QMA) [13]. Note: the error bars for reference ranges represent 1 standard deviation from the mean FREE2 methylation in blood of male controls (CGG < 44), PM males (56–170 CGGs), FM males with typical FXS (213–2000 CGGs) and 4 atypical ‘high functioning’ FM males unmethylated by Southern blot (UFM) (CGG 200–637 CGG) assessed as part of previous studies [13,21,22]. The reference samples were co-run with T1 and T2 DNA extracted from retrospectively retrieved newborn blood spots. AmplideX PCR targeting methylation of two *HpaII* sites, (**D**) HEX and (**E**) FAM channels from capillary electrophoresis of the DNA from the blood of the T2 male case in question. Note: pink background indicates region of >200 CGG repeats where presence of positive FM alleles with methylated *HpaII* sites and the control digestion (*HpaII* methylation independent) was detected.

Standard CGG sizing PCR on saliva DNA identified mosaicism of NS 30 CGG and PM 99 CGG alleles for T1, while for T2 only a PM 99 CGG allele was detected. Moreover, a potential peak in the area ~30 CGG repeats in T2 (Figure 1) could not be called a positive peak as it were below the threshold (200 relative fluorescence units (rfu)) for normal range alleles used for validated diagnostic testing using standard PCR at VCGS. In contrast a PM peak with a lower intensity (100 rfu) was called positive, as the positive threshold used for validated diagnostic testing at VCGS is different for alleles in the PM range at 50 rfu. Both mother (II-2) and maternal aunt (II-4) were identified to carry PM alleles of 109 and 138 CGGs, respectively (Figure 1). Follow-up diagnostic Southern blot testing confirmed the presence of a 109 CGG PM allele in the mother, while also identifying full mutation smears of 388 to 1025 CGGs in T1 and 672–1025 CGG smear in T2 (Figure 2). While presence of the PM 99 CGG allele was confirmed by Southern blot in T1, no NS allele was identified for T2. Southern blot testing did not identify presence of PM 99 CGG detected by standard PCR. There was no NS allele detected for T2 in either PCR or Southern blot analysis. The follow-up investigations using the Illumina CytoSNP300K (Illumina, San Diego, CA, USA), compared SNPs between the X chromosome, confirming 1st degree family inheritance, while also excluding presence of the second X chromosome in the male twins.

### 3.3. Follow-Up Investigation of Similarities and Differences between Twin Phenotypes

At 4 years and 11 months the twins were recruited into the FREE FX study, which involved collection of additional developmental and medical information and formal assessments of intellectual functioning and autism features. The clinical researchers who performed the psychological evaluations were blinded to the twins’ CGG class and DNAm status. Both twins were taking antipsychotic medications (Risperidone). In addition T2 was also taking Sertraline, a selective serotonin reuptake inhibitor. Medication regimen was not modified on the day of assessment.

The overall intellectual functioning of T1 as measured by the Wechsler Preschool and Primary Scale of Intelligence-3rd Edition (WPPSI-III) (Australian) was in the extremely low range, with a full scale IQ (FSIQ) of 62. The verbal IQ (VIQ) and performance IQ (PIQ) were in the borderline range (VIQ = 78) and extremely low range (PIQ = 55). All of Twin 2’s intellectual functioning scores fell in the extremely low range (FSIQ = 55; VIQ = 59, PIQ = 59). Of note is that neither child was able to obtain a valid processing speed score.

T2 was assessed with Module 1 of the Autism Diagnostic Observation Schedule-2nd edition (ADOS-2). This module is for children who are preverbal or using single words. During this assessment the child occasionally used phrase speech, but predominantly used single words. On the ADOS-2 the child had an overall comparison score of 6 meeting the ADOS-2 cut off for autism. Calibrated Severity Scores for the social affect and repetitive and restricted behaviours domains were 5 and 7, respectively. Significant behavioural issues were also observed during the ADOS-2 for T2 including hyperactivity, negative and disruptive behaviours and severe physically aggressive outbursts. This was also reported on the Aberrant Behaviour Checklist-Community by the mother. T1 was assessed with Module 2 of the ADOS-2 which is for children who are consistently using phrase speech. This twin also met the ADOS-2 cut-off for autism, and had a comparison score of 6. 

The social affect (SA) CSS was 6 and the repetitive and restricted behaviour (RRB) CSS was 7. While this child displayed some mild overactivity during the assessment it was not as significant as T2 and did not interfere with the assessment. No negative or disruptive behaviour was observed for T1 during the ADOS-2; though the mother also reported elevated maladaptive behaviours for T1, particularly on the irritability and hyperactivity domains, these were not as severe as T2. 

### 3.4. DNA Methylation Analysis

At the time of recruitment into the FREE FX study consent was also obtained for both twins for *FMR1* promoter methylation testing on retrospectively retrieved newborn blood spots, as well as the saliva samples collected at the time of initial investigation. *FMR1* promoter methylation testing involved analysis of the FREE2 consisting of 12 CpG sites located at the exon 1/intron1 boundary, previously associated with FMRP levels in blood [7], and intellectual functioning in males affected with FXS [20]. AmplideX mPCR testing was also used for follow-up CGG sizing and methylation analysis. 

While methylation percentage provided by this approach may not represent methylation of the larger *FMR1* promoter as it provides average methylation for only two *HpaII* methylation sensitive restriction sites (one within the *FMR1* CpG island and the other within *FMR1* exon 1) (Figure 2A), methylation of these sites has been shown to be elevated in FXS males [16]. Moreover, in contrast to FREE2 analysis, AmplideX mPCR methylation levels have not been previously significantly correlated with intellectual functioning or FMRP levels in cohort studies. However, this approach does provide methylation status for the *HpaII* restriction sites for different alleles, while FREE2 analysis provides information for the overall methylation levels.

FREE2 methylation analysis by two independent platforms (MALDI-TOF MS and MS-QMA) provided generally concordant results, with levels in both twins significantly elevated as compared to typically developing controls, PM and higher functioning unmethylated FM males (Figure 2C). The FREE2 methylation levels were between 74% and 84% in both twins, which approached mean methylation observed in the typical males with FM affected with FXS (Figure 2C). Interestingly, MALDI-TOF MS analysis showed methylation approximately 10% higher in T2 than T1. This suggests that less active retracted, but unmethylated alleles were present in T2 as compared to T1.

Subsequently, AmplideX mPCR analysis was conducted, however the volume of NBS DNA was not sufficient for follow-up, and testing was only performed on the remaining DNA from the saliva sample for T2. The T2 AmplideX mPCR results indicate that mosaicism was present for completely unmethylated NS and PM alleles and the 100% methylated FM allele, with the NS allele that was previously undetected by standard PCR testing. Furthermore, following AmplideX, mPCR resizing of T2 a PM allele was detected at 108 CGGs. This was discordant by nine CGGs to the size of the allele observed by standard PCR (Figure 1), while being very close to the size of 109 CGGs in the PM mother. While the difference in the PM allele size for T2 between standard PCR and AmplideX mPCR may be larger than anticipated, and may be technical in nature, there was also a smaller set of potential peaks detected in the 99 CGG range detected by AmplideX mPCR for the digestion control panel (Figure 2D). However, these were not called as positive, as they were below the recommended AmplideX mPCR positive threshold. Together, these results imply presence of partial methylated *FMR1* promoter, consistent with the FREE2 analysis, together with presence somatically unstable CGG size alleles ranging from normal to PM and FM size.

## 4. Discussion

This study presents a case of MZ twins affected with FXS with phenotypic and epigenetic discordant characteristics. Considering the molecular karyotype has excluded a second X chromosome and confirmed MZ inheritance, these differences may be explained through events that happen postzygotically. During early embryogenesis, cleavage of the blastomere can occur anywhere between weeks 1 and 2 and, during the time of the “splitting process”, the number of cells may not be evenly distributed to the future twins [18]. Consistent with this, this study demonstrated a case of MZ twins that inherited an unstable set of FM alleles that initially expanded from their mother’s PM 109 CGG allele, retracting somatically to leave both offspring with a mosaic nature of cell lines raging from unmethylated NS 30 CGG and PM 99 CGG alleles, to fully methylated FM alleles. The cause of the retraction is assumed to be an excision of the expanded CGG repeat potentially through CGG repeat replication slippage, although rarely flanking coding regions of the *FMR1* gene have been previously reported to be involved in the early post zygotic period producing the mosaic NS/PM and FM alleles [17,19].

While CGG size mosaicism for PM/FM alleles is not uncommon, it has been reported in up to 41% of FM males affected with FXS [23], and, to the best of our knowledge, this is the first report of MZ twins that are mosaic for NS, PM and FM alleles. This case is also the fourth report of a twin conception [12,24,25] that has demonstrated both expansion and retraction, which could also suggest a possible association between the twinning processes and PM allele instability.

While both twins in this study were severely affected with FXS, formal psychological assessments showed that T2 had much lower verbal intellectual functioning and more severe behavioural issues than T1. Specifically, it should be highlighted that T1 VIQ was 78, which is approaching the low average range (VIQ between 80 and 89), and eight points above cut-off of 70 (Average IQ of 100–2 standard deviations (SD = 15)). In contrast T2 had VIQ of 59, which falls in the extremely low range and is 19 points below T1’s score. This suggests that while both twins had FXS, they had clinically significant differences in their verbal abilities. Discordance for intellectual functioning has been also previously reported in monozygous females affected with FXS, where one female lived in an institution for handicapped, while another was ‘intellectually normal’ finishing high school, with FMRP levels consistent with the phenotype discordance [24]. However, unfortunately that study did not have formal assessments of intellectual functioning and behaviour available, including IQ scores, as presented here. 

Although, FREE2 methylation examined in this study has been previously correlated with FMRP in blood [14], in this study only DNA samples collected for diagnostic testing were available. This meant that *FMR1* mRNA and FMRP analyses could not be performed. Thus, other sample types should be collected in future studies to explore if the differences in DNA methylation observed between the twins reported in this study are related to *FMR1* mRNA and FMRP levels in different peripheral tissues that may be of close lineage to neurons, These could be hair root samples as previously described [24]. Moreover, the discordance in FREE2 methylation between the twins in this study was consistent with MALDI-TOF MS analysis that showed 10% higher methylation in T2. This was considered a biological difference as it was double the technical variability of ~5% (2 standard deviations), previously reported in replicate samples from 70 individuals described in Figure 1e of Cornish et al., 2015 [26]. This suggested that less active unmethylated alleles were present in T2 as compared to T1. This may not be sufficient to link the level of discordance in methylation to the differences in the phenotype without also showing differences in FMRP between the twins. It is also possible that another contributing factor is differences in the proportion of cells with normal size alleles that have resulted from somatic retraction, but may nevertheless become active. This is also consistent with the results from standard CGG sizing PCR, where a NS allele was not detected in T2 with more severe phenotype, potentially because it was present in a smaller proportion of cells than in T1. 

The NS retracted allele was unmethylated as confirmed by AmplideX mPCR, a potentially more sensitive test. The standard PCR is limited to size PM range to 120 CGG repeats; however, sensitivity limitations also did not detect the NS 30 CGG retracted allele from T2. This allele was unmethylated as confirmed by AmplideX mPCR, a technique which has a higher sensitivity for low abundance mosaic alleles than Southern blot. While Southern blot was performed to test for any expansions above the PM 99 CGG and 109 CGG allele sizes, it cannot detect mosaic cell lines if they are present in <20% [27]. This may explain why Southern blot did not detect the NS and PM alleles in T2, whilst the FREE2 MALDI-TOF MS methylation analysis showed that less than 20% of cells had unmethylated alleles for T2.

Despite the close correlations between the MS-QMA and MALDI-TOF MS platforms used in this and previous studies [13,28], there was a mild discordance between the twins for the platforms. MS-QMA methylation was almost identical between the MZ twins (Twin 1 71%; Twin 2 74%), while FREE2 MALDI-TOF MS showed methylation 10% higher for T2 (Twin 1 74%; Twin 2 84%). There are important technical distinctions that could explain the observed differences. Specifically, while both the MS-QMA and the MALDI-TOF MS analyse the same FREE2 region, within that region, the CpG3-5 cluster of fragments is too big in size (Daltons) to be captured by the mass spectrum methylation of these sites and cannot be captured by the MALDI-TOF MS system either (Figure 2A). In contrast, the aggregate measure of FREE2m from MS-QMA incorporates DNAm of CpG3-5 cluster, as MS-QMA does not employ mass spectrometry. Conversely, MS-QMA does not capture methylation of CpG1 which is included in DNA methylation analysis using the MALDI-TOF MS system.

## 5. Conclusions

This study demonstrates for the first time the clinical and molecular differences between 4-year-old MZ male twins mosaic for NS, PM and fragile X FM alleles with autism. It suggests that in genetically identical males with FXS, mosaicism due to postzygotic retraction may cause discordance in cell populations for CGG allele sizes and methylation patterns, which is related to the observed differences in verbal and intellectual functioning and severity of externalising behaviour problems. While the differences in the % of methylated alleles alone may not provide sufficient information to be directly beneficial in providing advice to this family in prenatal settings, the highlighted limitations between current diagnostic techniques in the mosaic scenarios reported in this study may have broader clinical implications. Specifically, the presence of an allele in the normal CGG size range in a male inherited from a PM female with no family history of FXS may not necessarily indicate that there is no FM present. FM, PM or normal size alleles may go undetected if present in a sufficiently small population of cells, as was the case for T1, where normal and PM alleles were not detected by Southern blot analysis, while being detected with a more sensitive PCR-based technique. Moreover, these technical differences have implications for interpreting FXS testing results, and may suggest that use of linkage analysis together with standard FXS testing, as previously described in another set of twins [12], may be beneficial in providing advice to the families regarding presence or absence of mosaicism for expanded and nonexpanded *FMR1* alleles, particularly in prenatal settings.

## References

[1] Hagerman R.J., Berry-Kravis E., Kaufmann W.E., Ono M.Y., Tartaglia N., Lachiewicz A., Kronk R., Delahunty C., Hessl D., Visootsak J. (2009). Advances in the treatment of fragile X syndrome. Pediatrics.

[2] Verkerk A.J., Pieretti M., Sutcliffe J.S., Fu Y.H., Kuhl D.P., Pizzuti A., Reiner O., Richards S., Victoria M.F., Zhang F.P. (1991). Identification of a gene (*FMR-1*) containing a CGG repeat coincident with a breakpoint cluster region exhibiting length variation in fragile X syndrome. Cell.

[3] Sutcliffe J.S., Nelson D.L., Zhang F., Pieretti M., Caskey C.T., Saxe D., Warren S.T. (1992). DNA methylation represses *FMR-1* transcription in fragile X syndrome. Hum. Mol. Genet..

[4] Brylawski B.P., Chastain P.D., Cohen S.M., Cordeiro-Stone M., Kaufman D.G. (2007). Mapping of an origin of DNA replication in the promoter of fragile X gene *FMR1*. Exp. Mol. Pathol..

[5] Willemsen R., Bontekoe C.J., Severijnen L.A., Oostra B.A. (2002). Timing of the absence of *FMR1* expression in full mutation chorionic villi. Hum. Genet..

[6] Eiges R., Urbach A., Malcov M., Frumkin T., Schwartz T., Amit A., Yaron Y., Eden A., Yanuka O., Benvenisty N. (2007). Developmental study of fragile X syndrome using human embryonic stem cells derived from preimplantation genetically diagnosed embryos. Cell Stem Cell.

[7] Godler D.E., Tassone F., Loesch D.Z., Taylor A.K., Gehling F., Hagerman R.J., Burgess T., Ganesamoorthy D., Hennerich D., Gordon L. (2010). Methylation of novel markers of fragile X alleles is inversely correlated with FMRP expression and *FMR1* activation ratio. Hum. Mol. Genet..

[8] Rodriguez-Revenga L., Madrigal I., Pagonabarraga J., Xuncla M., Badenas C., Kulisevsky J., Gomez B., Mila M. (2009). Penetrance of *FMR1* premutation associated pathologies in fragile X syndrome families. Eur. J. Hum. Genet..

[9] Kraan C.M., Bui Q.M., Field M., Archibald A.D., Metcalfe S.A., Christie L.M., Bennetts B.H., Oertel R., Smith M.J., du Sart D. (2018). *FMR1* allele size distribution in 35,000 males and females: A comparison of developmental delay and general population cohorts. Genet. Med..

[10] Biancalana V., Glaeser D., McQuaid S., Steinbach P. (2015). EMQN best practice guidelines for the molecular genetic testing and reporting of fragile X syndrome and other fragile X-associated disorders. Eur. J. Hum. Genet..

[11] Stark Z., Francis D., Gaffney L., Greenberg J., Hills L., Li X., Godler D.E., Slater H.R. (2015). Prenatal diagnosis of fragile X syndrome complicated by full mutation retraction. Am. J. Med. Genet. A.

[12] Prawer Y., Hunter M., Cronin S., Ling L., Aliaga Vera S., Fahey M., Gelfand N., Oertel R., Bartlett E., Francis D. (2018). Prenatal Diagnosis of Fragile X Syndrome in a Twin Pregnancy Complicated by a Complete Retraction. Genes.

[13] Inaba Y., Schwartz C.E., Bui Q.M., Li X., Skinner C., Field M., Wotton T., Hagerman R.J., Francis D., Amor D.J. (2014). Early Detection of Fragile X Syndrome: Applications of a Novel Approach for Improved Quantitative Methylation Analysis in Venous Blood and Newborn Blood Spots. Clin. Chem..

[14] Khaniani M.S., Kalitsis P., Burgess T., Slater H.R. (2008). An improved Diagnostic PCR Assay for identification of Cryptic Heterozygosity for CGG Triplet Repeat Alleles in the Fragile X Gene (*FMR1*). Mol. Cytogenet..

[15] Francis D., Burgess T., Mitchell J., Slater H. (2000). Identification of small FRAXA premutations. Mol. Diagn..

[16] Chen L., Hadd A.G., Sah S., Houghton J.F., Filipovic-Sadic S., Zhang W., Hagerman P.J., Tassone F., Latham G.J. (2011). High-resolution methylation polymerase chain reaction for fragile X analysis: Evidence for novel FMR1 methylation patterns undetected in Southern blot analyses. Genet. Med..

[17] Weschler D. (2004). Wechsler Preschool and Primary Scale of Intelligence—Third Edition Australian Standardised Edition.

[18] Lord C., Rutter M., DiLavore P.C., Risi S., Gotham K., Bishop S.L. (2012). Autism Diagnostic Observation Schedule, 2nd Edition (ADOS-2).

[19] Hus V., Gotham K., Lord C. (2014). Standardizing ADOS domain scores: separating severity of social affect and restricted and repetitive behaviors. J. Autism Dev. Disord..

[20] Arpone M., Baker E.K., Bretherton L., Bui M., Li X., Whitaker S., Dissanayake C., Cohen J., Hickerton C., Rogers C. (2018). Intragenic DNA methylation in buccal epithelial cells and intellectual functioning in a paediatric cohort of males with fragile X. Sci. Rep..

[21] Hwang T.Y., Aliaga S., Arpone M.V., Francis D., Li X., Chong B., Slater H.R., Rogers C., Bretherton L., Hunter M. (2016). Partially Methylated Alleles, Microdeletion and Tissue Mosaicism in a Fragile X Male with Tremor and Ataxia at 30 Years of Age: A Case Report. Am. J. Med. Genet..

[22] Hwang Y.T., Dudding T., Aliaga S.M., Arpone M., Francis D., Li X., Slater H.R., Rogers C., Bretherton L., du Sart D. (2016). Molecular Inconsistencies in a Fragile X Male with Early Onset Ataxia. Genes.

[23] Nolin S.L., Glicksman A., Houck G.E., Brown W.T., Dobkin C.S. (1994). Mosaicism in fragile X affected males. Am. J. Med. Genet..

[24] Willemsen R., Olmer R., De Diego Otero Y., Oostra B.A. (2000). Twin sisters, monozygotic with the fragile X mutation, but with a different phenotype. J. Med. Genet..

[25] Alfaro M.P., Cohen M., Vnencak-Jones C.L. (2013). Maternal *FMR1* premutation allele expansion and contraction in fraternal twins. Am. J. Med. Genet..

[26] Cornish K.M., Kraan C.M., Bui Q.M., Bellgrove M.A., Metcalfe S.A., Trollor J.N., Hocking D.R., Slater H.R., Inaba Y., Li X. (2015). Novel methylation markers of the dysexecutive-psychiatric phenotype in *FMR1* premutation women. Neurology.

[27] Aliaga S.M., Slater H.R., Francis D., Du Sart D., Li X., Amor D.J., Alliende A.M., Santa Maria L., Faundes V., Morales P. (2016). Identification of Males with Cryptic Fragile X Alleles by Methylation-Specific Quantitative Melt Analysis. Clin. Chem..

[28] Godler D.E., Inaba Y., Schwartz C.E., Bui Q.M., Shi E.Z., Li X., Herlihy A.S., Skinner C., Hagerman R.J., Francis D. (2015). Detection of skewed X-chromosome inactivation in Fragile X syndrome and X chromosome aneuploidy using quantitative melt analysis. Exp. Rev. Mol. Med..

